# Subcontracting sterile pediatric and adult chemotherapy preparations activity: A global risk analysis

**DOI:** 10.1002/hsr2.571

**Published:** 2022-06-20

**Authors:** Julia Rousseau, Kaouther Zribi, Camille Cotteret, Ambroise Marcais, Sarah Winter, Gabriel Salguero‐Hernandez, Salvatore Cisternino, Joël Schlatter

**Affiliations:** ^1^ Pharmacie, Hôpital Universitaire Necker—Enfants Malades Assistance‐Publique des Hôpitaux de Paris Paris France; ^2^ Service d'Hématologie Adulte Assistance‐Publique des Hôpitaux de Paris Paris France; ^3^ Service d'Immunologie, Hématologie et Rhumatologie pédiatrique, Hôpital Universitaire Necker—Enfants Malades Assistance‐Publique des Hôpitaux de Paris Paris France; ^4^ Imagine institute INSERM U1163 Paris France; ^5^ Optimisation Thérapeutique en Neuropsychopharmacologie, INSERM UMR_S1144 Université de Paris Paris France; ^6^ Pharmacie, Hôpital Paul Doumer Assistance‐Publique des Hôpitaux de Paris, AP‐HP Labruyère France

**Keywords:** adult, global risk analysis, peadiatric, risk management, sterile chemotherapy preparation, subcontracting

## Abstract

**Objective:**

The main purpose of this study was to carry out a global risk analysis (GRA) on the subcontracting circuit to determine and evaluate the risks linked to the future subcontracting process and to propose corrective actions for the most critical risks to ensure safety. This study must allow to conclude in an objective way to the feasibility or not of this project.

**Methods:**

A GRA was performed, conducted by a multidisciplinary working group that met in 20 meetings, corresponding to about 50 h of work.

**Results:**

We identified 92 scenarios: 13% of scenarios had an initial criticality C1, 40% C2, and 47% C3. The GRA shows that the riskiest scenarios concern the management, material, and equipment with IT system and logistics with transport. The working group identified 25 corrective actions. After implementing those actions, 85% of scenarios had residual criticality C1, 8.5% C2, and 6.5% had residual criticality C3. The working group chose that it was impossible to subcontract part of the activity.

**Conclusion:**

The GRA conducted in this study highlighted the risks related to outsourcing this activity, evaluated and prioritized them, and recommended corrective actions. Therefore, we conclude that subcontracting the totality of sterile preparations would be harmful to patient care quality and reactivity for vital medical emergencies, such as macrophage activation syndrome, preparation of clinical trials, graft rejection therapies, preparation of very short stability chemotherapy, and the pediatric graft conditioning chemotherapy.

## 1 INTRODUCTION

Over the last 10 years, the French public hospital debt tripled to achieve approximately 30 billion euros.^1^ To address the failure of health cost control, French governmental institutions have implemented a radical performance reform. Where complete and unrestricted patient management predominated, effective cost‐management performance was essential to balance budgets. Therefore, in a restrictive financial situation and with the human resources redeployment, outsourcing seems to be a way of allowing hospital operations to concentrate on medical care by contracting out services, including equipment maintenance, biological cleaning, and medico‐technical operations.^2^ Today, institutions aim to extend subcontracting to high‐risk areas such as sterile chemotherapy production Guimarâes and Crespo de Carvalho.^3^ Indeed, the production of sterile chemotherapy preparations requires many staff and expensive technological equipment. Outsourcing the production would reduce risks linked to low production, allocate time gained on pharmaceutical tasks, absorb costs involved in this activity, upgrade technical skills as well as redistribute staff to other areas of the order site Galy et al.^4^ Following these costs arguments, our hospital management suggested outsourcing the pediatric and adult sterile chemotherapy preparation activity to another hospital. Currently, our hospital prepares chemotherapies, particularly for a pediatric department performing hematopoietic stem cell transplants for patients with immune deficiencies and chemotherapies for the adult onco‐hematology departments with conventional and intensive care. Some preparations are also made for other pediatric services, such as pediatric nephrology, pediatric intensive care, and medical‐surgical continuing care units, neurology, and some adult services like surgical intensive care, nephrology, and multidisciplinary day hospital. To determine and assess potential hazards related to outsourcing the sterile chemotherapy preparations, the global risk analysis (GRA) was selected in our hospital to lead the a priori analysis. The GRA method is derived from the preliminary risk analysis created initially in 1950s for the aerospace and military departments and widespread to industries. Adapted to health, the GRA can define the consequences from the causes and corrective actions to be implemented.^1^ This study is designed to provide a fully objective conclusion on the applicability of the outsourcing project.

## 2 METHODS

### 2.1 Sterile centralized preparation for injection unit (CPIU)

Eighteen 149 sterile preparations were realized in 2018; about 50% were chemotherapy and the others were injectable preparations for pediatric use specifically (e.g., defibrotide, antivirals, antifungals). The CPIU is opened Monday to Friday from 9 a.m. to 6 p.m., and Saturday 9 a.m. to 1 p.m. Physicians prescribed chemotherapies on Chimio® software (published by Computer Engineering, version 5.8). For the patients treated in the outpatient clinic, prescriptions are made 24 h before administration (Day 1). For inpatients, prescriptions can be made the day before or on the administration day (Day 0). The pharmacist reviews and approves the prescription. For this purpose, he/she consults the patient medical file to check the correlation between the treatment prescribed and multidisciplinary consultation meeting decision. The height and weight data, biological assessment, compliance with the intercure, and tolerance to treatment during previous cures are verified too. For short stability preparations, production is performed on the day of administration. For expensive drugs, although the stability of the drug permits to anticipate the preparation, the CPIU waits for the doctors “GO/NO GO” to prepare the chemotherapy (e.g., carfilzomib, brentuximab). Our hospital is also an approved JACIE center (joint accreditation committee of international society for cellular therapy and European bone marrow transplantation). It is accredited by a European organization specialized in hematopoietic stem cell transplantation (HSCT). It is developing an international accreditation system to ensure high quality of patient care and improve the performance of specialized centers in the collection, management, and transplantation of SCT (European Society for Blood and Marrow Transplantation [EBMT], 2021).^5^ The SCT transplant activity is performed in the adult hematology departments and in the pediatric immunohematology department. In 2018, the hospital performed about 100 transplants for both pediatrics and adults.

### 2.2 Implementation of GRA

**Figure 1:**
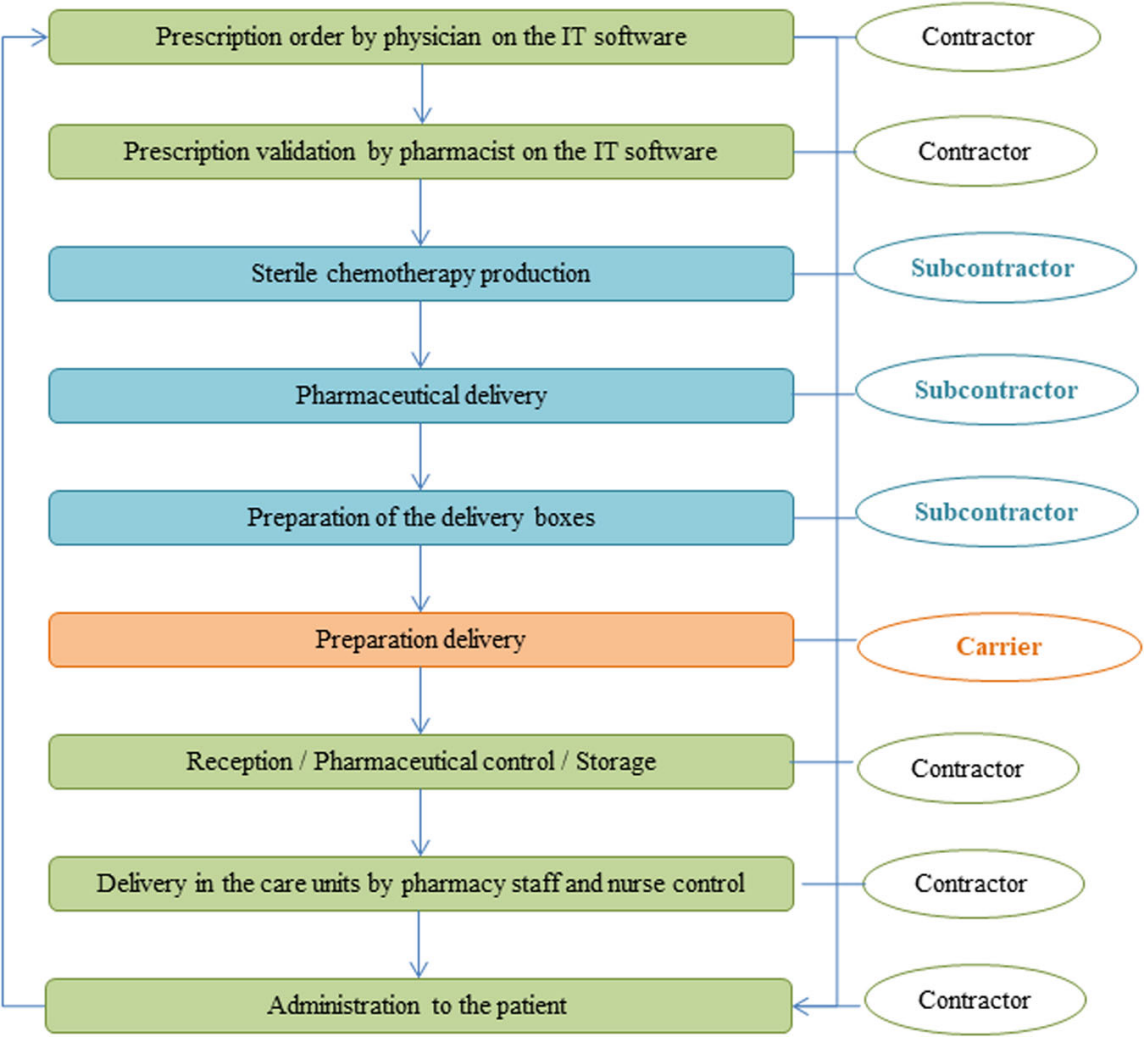
Outsourcing process of sterile chemotherapy preparations

Analyses were conducted by a multidisciplinary working group from December 2018 to May 2019, consisting of two physicians (pediatric and adult hematologists), pharmacists in charge of the sterile preparation unit, pharmacists familiar with the methodology, a resident, three pharmacy technicians, and nurses. The group worked without any hierarchy and decisions required a common agreement. The workgroup identified the proposed outsourcing circuit (Figure 1).

### 2.3 GRA method

The GRA method consists of two sequential steps: GRA system and GRA scenario. The aim of the GRA system is to define the scope of the system under study and to identify all hazardous situations that the system could potentially encounter when contracting out sterile chemotherapy preparations Desroches et al.^6^ For this task, the outsourcing process was divided by the working group into five main steps (prescription, sterile chemotherapy preparation, delivery, preparation receipt, and administration to the patient), 6 phases, and 10 subphases (Table 1). The hazards list was established similarly to the one proposed by the Ecole Centrale Paris and adapted to our practice.^1^ Finally, 8 generic hazards, 12 specific hazards, and 21 hazardous events have been selected (Table 2). By cross‐linking hazards and subphases, hazardous situations cartography was built. Each interaction between a hazard and a subphase was evaluated by assigning a score using a scale defined by the working group. This scale categorizes risks into two levels of priority such as high priority (Priority 1), the situation is considered as dangerous and needs to be assessed immediately and low priority (Priority 2), interaction is weak and the situation is safe. The working group focused only on the Priority 1 situations.

**Table 1:** Outsourcing circuit depicted in steps, phases, and subphases

Steps	Phases	Subphases
1. Prescription (contractor)	Prescription on the IT software	Patient inclusion on the IT software by the physician
		Validation of the prescription validation on the IT software by the physician
	Analysis and pharmaceutical validation	Validation of the prescription on the IT software by the pharmacist
2. Sterile chemotherapy preparation (subcontractor)	Production	Production and liberation of the preparation
3. Delivery (private contract)	Delivery of preparations	Carrying preparations
4. Preparation receipt (contractor)	Preparation receipt	Receipt of the preparations
		Control the preparations
		Storage preparations
		Delivery preparations to the care units
5. Administration to the patient (contractor)	Administration to the patient	Administration to the patient

**Table 2:** Hazards related to sterile chemotherapy preparation outsourcing

Generic hazards	Specific hazards	Hazardous events
Environmental	Natural	Adverse meteorological conditions
Management	Organization	Failure to organize
		Inaccurate estimation of the future activity
		Tasks interruption
	Workforce resources	Insufficient training
		Failure to recruit
		Non‐qualified personnel
Communication	Organization	Lack of coordination between teams
	Support	Lack of formalization
Politic and regulation	Institutions	Drug with a restricted status (clinical trials…)
Infrastructure	Premises	Inadequate space
		Lack of security
Material and equipment	Material and equipment in operation	Malfunction of the system
		Absence of harmonization of IT equipment
	IT system	Defective network system
		Software failure
Human factor	Individuals	Stress, fatigue, lack of motivation
		Resistance to change
Logistic	Gestion	Management defect of restricted status drugs
	Delivery	Transport safety defect
		Traffic hazards (accidents)

The GRA scenario is the second methodology step. It assesses any consequences caused by the hazardous situation and determines the corrective actions required to secure the system. The working group developed scenarios related to each hazardous situation. Each scenario was evaluated according to criticality to prioritize the implementation of corrective actions. For this purpose, severity (S) and frequency (F) scales were defined (Table 3). The crossing between severity and frequency index generates a criticality (C) index C = S × F, defined by the criticality matrix. Three classes of criticality were identified: C1: acceptable, C2: tolerable under control, and C3: unacceptable (Table 4). Thus, the ranking of each scenario according to the criticality matrix generated the initial risk mapping. For the scenarios rated C3 and C2, corrective actions were proposed by the working group. These corrective actions have been classified by priority based on risk level and the expected effort to minimize the risk. The residual risks of each scenario were evaluated and rated again with criticality indices considering the actions decided upon. If unacceptable scenarios persisted, residual risks were treated by the implementation of additional corrective actions or discontinuation of some activities. All analyses were conducted using the software StatCart® (V2.0; MAD‐Environnement).

**Table 3:** Severity and frequency scales

Severity scale		
S1	Minor	**None or low impact on business performance and safety**
		No impact on the patient Delay in production <1 h No financial damage Minimal exposure to chemical risk and protection of the individual No impact on staff
S2	Significant	**Degradation of system performance without impact on safety**
		Management delay <2 h without impact on the patient Half‐day production delay Financial loss <10,000€ Discharge on a limited and protected area Low impact on staff (e.g., stress)
S3	Major	**High degradation or failure of system performance without impact on safety**
		Management delay >2 h without impact on the patient One day production delay Financial loss between 10,000€ and 100,000€ Discharge of a large volume and protected individual Moderate impact on staff Degradation of system integrity
S4	Critical	**Degradation of system integrity**
		Management delay >2 h with impact on the patient (e.g., pediatric busulfan) or life‐threatening prognosis Subcontracting temporarily stopped Financial loss between 100,000€ and 300,000€ Major discharge without projection and unprotected individual High impact on staff (e.g., temporary work stoppage)
S5	Catastrophic	**Significant degradation or total deterioration of system integrity**
		Permanent invalidity or death of the patient Permanent cessation of outsourcing Financial loss >300,000€ Major discharge and projection on unprotected individual Catastrophic impact on staff (e.g., permanent work stoppage)
**Frequency**		
F1	Exceptional	Less than once a year
F2	Very improbable	Between once a year and once every 6 months
F3	Improbable	Between once every 6 months and monthly
F4	Probable	Between monthly and weekly
F5	Very probable to certain	More than weekly

**Table 4:** Criticality matrix

		Severity				
		S1	S2	S3	S4	S5
**Frequency**	F5	C2	C3	C3	C3	C3
	F4	C1	C2	C3	C3	C3
	F3	C1	C2	C2	C3	C3
	F2	C1	C1	C2	C2	C3
	F1	C1	C1	C1	C1	C2

*Note*: C1, the risk was considered acceptable; C2, the risk was considered tolerable under control; C3, the risk was considered unacceptable.

## 3 RESULTS

### 3.1 GRA system

**Figure 2:**
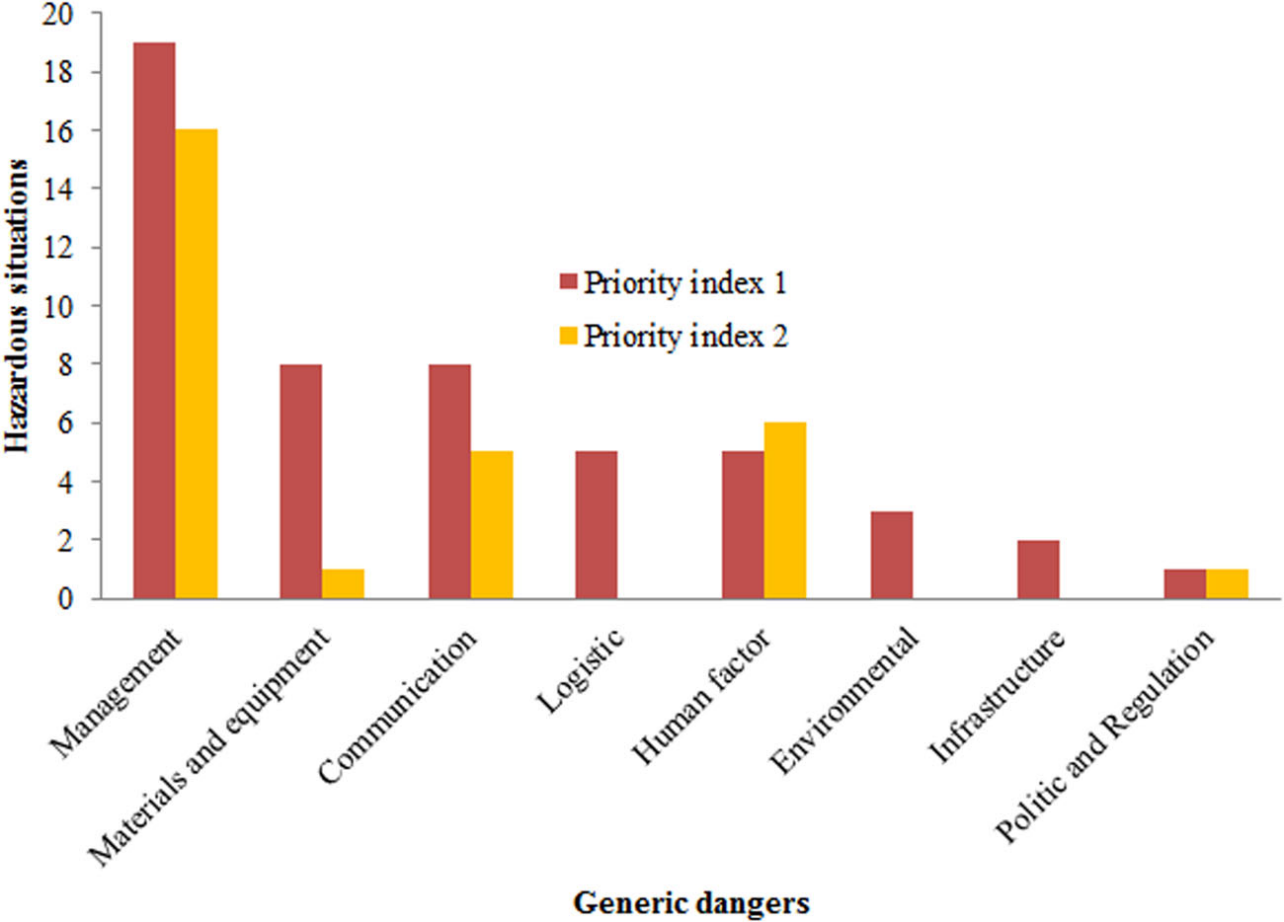
Priority indexes of hazardous situations by generic hazards

The working group identified 80 potential hazardous situations including 51 with priority 1 to be treated immediately and 29 with priority 2 to be analyzed later. Focusing on high priority situations, management (37%) with organizational failure and insufficient training of staff, materials and equipment (16%), communication (16%), delivery (10%), and human (10%) were the most relevant generic hazards (Figure 2).

### 3.2 GRA scenario

**Figure 3:**
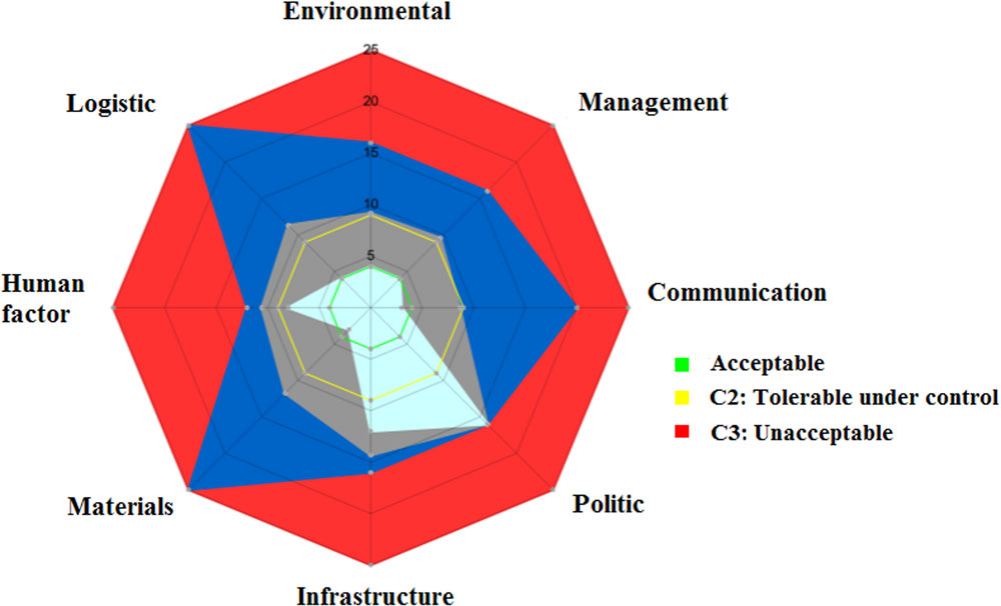
Chart radar of initial dangers

**Figure 4:**
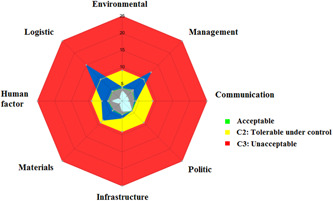
Chart radar of residual dangers

The 51 hazardous situations generated 92 scenarios. Twelve scenarios (13%) had an initial criticality C1, 37 (40%) C2, and 43 (47%) C3. Regarding initial dangers on the radar chart, logistics and material and equipment were the major dangers on the system (Figure 3). Following corrective action, the majority of system dangers remained tolerable under control, but management and logistics remained unacceptable (Figure 4). The working group identified 25 corrective actions (Table 5). After implementing these corrective actions, 78 scenarios had residual criticality C1 (85%), 8 (8.5%) C2, and 6 (6.5%) had residual criticality C3, unacceptable, despite the risk reduction plan.

**Table 5:** Depiction of the 25 corrective actions

	Corrective action	Step	Content	Responsible	Commentaries
1	To contract the outsourcing activity	All the system	Prioritization of the preparations of the contractor and subcontractor Management of urgent prescriptions by the contractor Management of costly preparations by the contractor. Communication with departments about planning changes Defining the time ranges of prescriptions Setting of the number of trips and delivery per day Management of urgent trips. Anticipating prescriptions 48 h in advance Establishing the production hours of the contractor Management of the demands out of working days Establishing the legal responsibilities of each party	Directors of the hospitals Pharmacists responsible in the sterile chemotherapy preparation activity of the hospitals	Maintaining a residual activity in contractor hospital for the vital emergency <3 h, short‐term expiry date, transplant packaging specifications
2	To define the terms of transport	Delivery Reception	Provide an alternative route if traffic is impossible To define the procedures for the reception of the preparations Check the conformity of the boxes on departure and arrival (sealing, storage conditions) Anticipate the acquisition of equipment (isothermal transport boxes, temperature monitors) Allow two temperature monitors per isothermal box	Financial directors Pharmacists	Extra costs generated by the acquisition of equipment
3	Formalize the maximum delay for the results of the busulfan adaptation	Prescription	To impose time limits for reporting pharmacokinetic results of the pediatric busulfan dose adjustment	Physicians Biologists	Maintaining the pediatric transplant activity on the contractor site
4	Training sufficient number of pharmacists in the creation of protocols on the contractor site	Pharmaceutical Validation	Establish a planning for the attendance of trained pharmacists to create protocols	Pharmacists	Draft a comprehensive procedure for the creation and validation of a protocol Involve the subcontractor's pharmacists in protocol validation
5	To contract the delivery of preparations between the pharmacy ordering and the healthcare departments	Reception	Draft a procedure with the attendance times of the messengers For prescriptions made after the deadline, determine the collection of preparations by the departments Inform the department upon reception and control of the preparations	Healthcare managers	Inform the guard if there is a delivery problem
6	To control boxes from the subcontractor site	Delivery	Purchase transport module of the software Using a computer system such as a barcode Using a color code on boxes based on the destination site	Pharmacists	Extra costs associated with equipment acquisition
7	To modify the offices of the contractor	Reception	Control preparations in an independent and specific room Designate specific storage rooms To delimit and define a place for the possible isolation of preparations	Construction manager	Risk of inadequate assessment of locations
8	To train the personnel of the subcontractor site to the new activity	Production	Training the personnel of the subcontractor site to the new protocols and their specific requirements	Pharmacists	Difficulties in maintaining training, in implementation and evaluation Posttraining assessment and audit Training and authorization file
9	To train the staff of the contractor site in the new procedures	Reception	Training in receipt (monitoring of temperature curves), inspection, storage conditions of preparations in the specific place To define accurately the role of each actor in the reception of a delivery	Pharmacists	Training and authorization file
10	To train staff in CMR (carcinogenic, mutagenic, reprotoxic) risks	Reception Delivery	CMR risk training Training carriers in CMR risks. Provide a cytotoxic emergency kit in each truck	Pharmacists	Training and authorization file
11	To reorganize the pharmaceutical activity of the contractor	Pharmaceutical Validation	Planning of pharmacists qualified to validate prescriptions on the software considering possible unanticipated absences. Alternating between the pharmacists who validate and those who control the preparations	Pharmacists	Education of all pharmacists
12	To define the need for human resources	Production	Matching the activity with human resources	Directors Pharmacists	Negotiation with the management department
13	To define the need for human resources for transport	Delivery	Determine a minimum number of transporters considering unplanned absences	Logistic director	Negotiation with the management department
14	To contract the management of specific status drugs	Production	Procedures for the prescription of drugs under specific management (clinical trials…) Contracting roles for each party	Pharmacists	
15	To set a downgraded process	Prescription	Manually transmitted prescription to the subcontractor in case of network failure	Pharmacists	Prescription with a higher risk of error than software prescription Dual pharmaceutical control of prescription
16	To set a downgraded process	Production	Defining the backup production if the contractor site is not operational Downgraded procedure of the production sheet	Pharmacists	Preparation with a higher risk of error Dual pharmaceutical control of production sheet
17	To secure the storage in the healthcare departments of the contractor site	Administration	Set up probes for temperature registration in each refrigerator	Healthcare managers	
18	To harmonize the computer bases between the two sites	Prescription Production	Harmonizing protocols for both sites Using the same version of the software Unique database	Pharmacists Physicians IT experts	Problems in obtaining professional consensus Setting up a working group
19	To plan a training program for staff to accompany them through changes	All the system	Briefing staff on the new organization Proposing a plan for restructuring human resources. Assisting each job transition beforehand	Management directors	Danger of work interruption
20	To train the prescriber of the contracting site in the new organization	Prescription	Formation of the prescribers on the prescription schedules, the importance of communication with the pharmacy, the procedures linked to subcontracting	Physicians Pharmacists	Inadequate number of trainers to provide training Heavy task E‐learning on the operational conditions of subcontracting
21	To sensitize the patient about the outsourcing	Administration	Propose a video on the outsourcing circuit from prescription to administration Draft an information brochure on the circuit for each patient	Healthcare managers Pharmacists Physicians	
22	To consolidate network if necessary	Prescription Production Administration	Diagnosing the network	IT experts	Potential network failure
23	To relocate clinical trial logistics of the contractor	Prescription Production	Management of the contractor's clinical trials by the subcontractor (reception, storage, preparation, dispensation, and traceability)	Pharmacists	Risk of a reduction in the inclusions of the contractor's patients
24	To maintain the clinical trials activity on the contractor site	Prescription Production	Continuation of management of clinical trials	Pharmacists	
25	To contract the management of short‐term stability drugs	Production Administration	Providing urgent delivery for short‐stability preparations	Directors Pharmacists	Surcharge for extra trips Excessive time required for availability Unacceptable risk to administer an expired preparation Sustaining production at the contractor site

## 4 DISCUSSION

Outsourcing one or several hospital activities operations is a common initiative that started many years ago in France and worldwide. Nevertheless, little data is available on the positive or negative economic, social or clinical impact. Because outsourcing pharmaceutical operations, such as sterile chemotherapies preparation, leads to patient safety implications, implementing GRA provides an appropriate systemic approach by mapping dangerous situations Mazeron et al.^7^ The GRA prioritizes suitable measures and efforts to ensure the safety of the system by taking corrective actions. The residual risk mapping performed upon completion of the analysis can evaluate the impact of these corrective actions. The GRA study conducted on outsourced sterile chemotherapy preparations showed that the whole system had residual critical hazards.

### 4.1 Logistic

Transporting carcinogenic, mutagenic, or toxic for reproduction compounds is highly hazardous and implies training and certification of personnel for the task. Every transport vehicle must be equipped with a decontamination kit in case of an accident and the deliverer should be certified to manage a hazardous chemicals accident. Selection of suitable equipment (isothermal packaging, thermal probes, refrigerated vehicles, etc.) will be essential to guarantee the integrity of preparations in transit. The supplier should comply with criteria, such as the packaging specifications (number of bags per box, sealed containers, etc.) and storage conditions. Periodic audits of the shipping company must be performed by the contracting institution. In addition, traffic risks and long delays can be a deterrent to outsourcing these preparations.

### 4.2 Materials and equipment: IT system

The working group recommended the development of one electronic database for both sites Rucheton et al.^8^ Achieving medical consensus for a single thesaurus for both sites could be long and difficult. In the event of software failure or computer network breakdown, a downgraded mode protocol should be established.

### 4.3 Human factor

Every new work organization can affect staff with the risk of high resistance to change. Indeed, opposition against change could be a major cause of social conflict. Changes in management typically result in redeployment or reconversion with a direct impact on work and private life. An article about the impact of outsourcing on workers shows that outsourcing can pose a moral challenge to workers (Rousseau, 2016).^9^ Outsourcing attractive work challenges professional expertise with a sense of devaluation of the tasks they do. It may create an inefficient division of staff in the two locations, with a risk of competition between the teams affecting cooperation. Information and professional assistance before subcontracting implementation are therefore very important. A preliminary risk analysis of the temporary subcontract of the sterilization activity revealed the outsourcing impact on staff work patterns Alméras et al.^10^ The study pointed out a need to work extra hours with constant staff, an increased workload over a shorter period of time, significant handling related to transport, more rigor due to the precautions required during the sending and receiving phases, and an impact on transport including adjustments in the shuttle schedules initially planned.

The subcontracting process must be founded on an evaluation of the social risk, competition between service providers, financial and tax impact, and quality control of the services supplied. Subcontracting implies a general reorganization of the ordering center. The human factor of resistance to change is too often underestimated.

### 4.4 Training at both sites

Staff training at the subcontractor's site is critical for chemotherapy preparation, particularly for pediatric protocols. The pediatric population has been identified as at risk for serious adverse events.

Indeed, “the overdose of anticancer drugs, especially paediatrics“ is one of the nerver events of the National Agency for the Safety of Medicines and Health Products's list in France.^11^

### 4.5 Activities classified as high risk to outsource

For short stability drug preparations (<4 h), it is almost impossible to provide them in time. Administration of an outdated formulation or a delay in patient care was a major risk. For example, the melphalan preparation is stable for 90 min between reconstitution and the end of administration European Medicines Agency.^12^ Intrathecal preparations are at high risk, therefore stability was set at <4 h in our hospital. Preparation of chemotherapies for vital emergencies may constitute an obstacle for outsourcing if it takes too long to get the preparation ready for clinical service. Macrophage Activation Syndrome (MAS) is a fatal complication of hematological diseases requiring immediate and appropriate treatment Imashuku et al.^13^ Hematologists advise the administration of etoposide within 3 h of diagnosis. The subcontractor must be able to satisfy the request on working days as well as on evenings, nights, weekends, or public holidays. Although SAM is a rare disease, AGR recommended internal organization be maintained to ensure a safe response for the patient. The chemotherapy reconstitution unit also makes sterile intravenous (IV) preparations for some clinical trials. For these particular sterile preparations, the administrative management (inclusion in the clinical trial, allocation of vials…) can rarely be predicted and can delay the administration of treatment to the patient if subcontracted. The working group advises maintaining the management of the sterile IV preparations linked to clinical trials at the contractor site.

**Figure 5:**
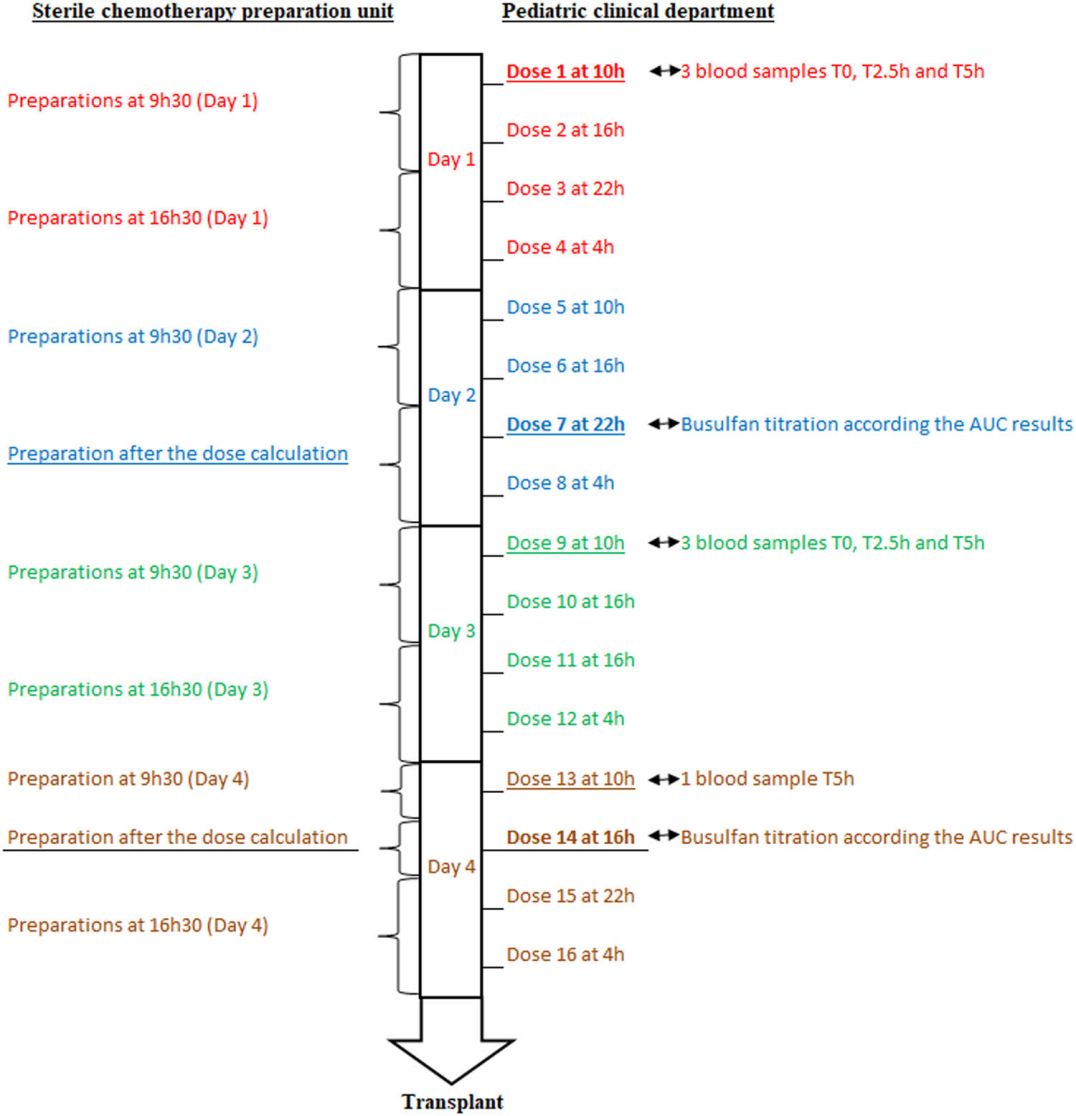
Typical myeloablative protocol with busulfan for stem cell transplantation

Our unit prepares chemotherapies for HSCT, mainly in pediatrics for immune disorders. In the pediatric area, busulfan is used in a sophisticated schedule as myeloablative chemotherapy preceding autologous or allogeneic HSCT. The organization of the UCPI for busulfan preparation (Figure 5) depends on its stability (12 h).^14^ IV administration of busulfan consisted of a fixed infusion of 3 h every 6 h for 16 doses. Busulfan doses are titrated in children based on their area under the curve (AUC) after the first dose. The pharmacokinetic dosage of IV busulfan is critical as low AUCs may cause graft failure while high AUCs are related to severe toxicities. In this protocol, IV busulfan doses are titrated to dose 7 and dose 14 depending on the results of AUCs. Delays in pharmacokinetic results due to incorrect collection (inadequate storage and faulty collection schedule) or other reasons could reduce the time from preparation to patient administration. It is common for the prescription of dose 14 based on AUC results to be completed 30–60 min before the scheduled time of administration. Outsourcing delays may compromise the success of the conditioning and transplant process. Considering all these data, the working group estimated the risk of outsourcing the preparation of busulfan at a rate too high for patients and recommends that the activity remains in our hospital.

### 4.6 Impact of a pandemic on our system

We need to adapt our risk management strategy during pandemics, such as the one we are currently experiencing with SARS‐Cov‐2. Indeed, we may be confronted with both understaffing in case of sick people or with childcare issues related to containment, and impact on the production and delivery of raw materials (e.g., drugs and medical devices) required for the production of chemotherapy.

To strengthen the resilience of the system, it is necessary to favor a sufficient number of people trained to produce chemotherapy by limiting the decrease in the number of production units.

Indeed, SARS‐Cov‐2 has made the management and care of cancer patients more complex, subcontracting adds to this an organizational constraint for the care services and pharmacy of the ordering site, which have to adapt to the work schedules of the provider site but they are not always compatible.

In addition, the logistical flow inherent in subcontracting requires additional human resources and extends patient care time in the event of a therapeutic emergency that may arise in certain malignant onco‐hematological diseases.

These constraints can be avoided by promoting production units closer to patients, allowing better control of the continuity, resilience, and responsiveness of care.

### 4.7 Study limits

Pre‐existing risk analyses have been performed in the area of health care, but data in the literature on contracting out centralized injectable preparations are scarce.^15^, ^16^, ^17^, ^18^, ^19^, ^20^, ^21^ Therefore, it was difficult to compare with other studies to improve our GRA. The implementation of the GRA requires significant time and effort. Its complexity lies in the establishment of a multidisciplinary working group and the numerous meetings necessary to make progress on the study. It was especially challenging for all participants to be present at each meeting. The absence of financial impact evaluation of sterile chemotherapy preparation outsourcing is an additional limit. There are fewer data available in the literature about outsourcing costs. In a study performed to determine the interest in outsourcing the sterilization process, authors conducted an economical assessment of in‐house sterilization, outsourcing sterilization to an industrial firm, and outsourcing sterilization to another healthcare establishment Tigahig et al.^22^ Results revealed an estimated cost of 251,647 euros for in‐house sterilization, 505,599 euros for outsourcing to the industrial company, and 541,635 euros for outsourcing to another healthcare establishment. The actual cost of outsourced activity should therefore be systematically evaluated. A recent study about the costs of subcontracting the chemotherapy preparations revealed that the loss of preparations was linked to premature medical prescription, miscommunication, and prescription errors Petrovic et al.^23^ Therefore, 71% of the losses were avoidable. The analysis of these data motivates the presence of a pharmacist in the care unit to optimize communication between doctors and the pharmacy.

### 4.8 Risk management plan

The overall effort induced by the reduction plan was considered important. This could represent an additional cost to be assessed. In addition, certain risks were not entirely under control. For six scenarios, the residual criticality remained unacceptable. No appropriate action was identified to manage their risks.

The working group agreed not to externalize the preparation of short stability drugs, drugs indicated in vital emergency situations (macrophagic activation syndrome), preparations for clinical trials, and the pediatric preparation of busulfan. For such situations, we proposed to maintain a residual activity at the ordering center; it results in a cost related to the use of the unit. To measure the quality of subcontracting, indicators of control and performance will be implemented. The indicators refer to ongoing staff training with annual assessment, registration of nonconformities at the various stages in the new circuit, tracking of emergency prescriptions and their management, delays in transporting preparations, and the losses generated by subcontracting (destruction of preparations, need to extend hospital stays, etc.). The indicators should enable feedback on adverse incidents that occurred and new actions for reducing risks to secure the subcontracting circuit. An overview study on the indicator data after chemotherapy subcontracting identified 56 nonconformities Merouani‐Bouhbouh et al.^24^ this concerned information system (computer failure), preparation phase (solvent error), storage errors with wrong thermal probes, and destination errors. A circuit adaptation was required to reduce delivery delays and preparation destructions caused by early production (3% of lost preparations).

## 5 CONCLUSION

The outsourcing of adult and pediatric sterile preparations to improve the quality, safety, and efficiency of the system should not be performed in the absence of risks analysis associated with this new organization. The GRA conducted in this study highlighted the risks related to outsourcing this activity, evaluated and prioritized them, and recommended corrective actions. Therefore, we conclude that subcontracting the totality of sterile preparations would be harmful to patient care quality. In that case, we should maintain a residual activity with costs associated.

The decision involved vital medical emergencies with a duration of under 3 h, such as MAS, the preparation of clinical trial chemotherapy, the preparation of very short stability chemotherapy, such as melphalan (<90 min), intrathecal chemotherapy, and the pediatric graft conditioning chemotherapy. Moreover, major efforts were deemed indispensable to harmonize the databases of both sites and the implementation of staff training. Monitoring indicators were suggested by the working group: nonconformity monitoring related to the subcontracting circuit, tracking urgent unforeseen prescriptions, the delay between the prescription order and the delivery in the department, monitoring of non‐conformities linked to the computer network, tracking returns and preparation losses, and delivery time monitoring.

## AUTHOR CONTRIBUTIONS

**Julia Rousseau**: conceptualization; funding acquisition; investigation; methodology; validation. **Kaouther Zribi**: conceptualization; formal analysis; methodology; supervision. **Camille Cotteret**: conceptualization; data curation; supervision; writing – review and editing. **Ambroise Marcais**: validation; writing – original draft; writing – review and editing. **Sarah Winter**: validation; visualization; writing – review and editing. **Gabriel Salguero‐Hernandez**: funding acquisition; resources; supervision. **Salvatore Cisternino**: validation; visualization; writing – review and editing.

## CONFLICTS OF INTEREST

The authors declare no conflicts of interest.

## ETHICS STATEMENT

Not applicable.

## Data Availability

The data are available on request from the corresponding author
